# The Influence of Educational Level on the Perception of Altered Smile Esthetics Among Dental Students: A Cross-Sectional Study

**DOI:** 10.3390/dj13070287

**Published:** 2025-06-25

**Authors:** Panagiotis Ntovas, Ioulianos Rachiotis, Panagiotis Maniatakos, Nikolaos Loumprinis, Chariklia Paximada, Christos Rahiotis

**Affiliations:** 1 Department of Operative Dentistry, Dental School, National and Kapodistrian University of Athens, 11527 Athens, Greece; pan.ntovas@gmail.com (P.N.); chpaximada@gmail.com (C.P.); 2 Department of Prosthodontics, School of Dental Medicine, Tufts University, Boston, MA 211, USA; 3 Division of Fixed Prosthodontics and Biomaterials, University Clinics for Dental Medicine, University of Geneva, CH1211 Geneva, Switzerland; 4 Dental School, National and Kapodistrian University of Athens, 11527 Athens, Greece; juliorah@gmail.com (I.R.); panagiotismaniatakos@gmail.com (P.M.)

**Keywords:** smile perception, dental education, smile esthetics, dental students, smile attractiveness

## Abstract

**Background/Objectives**: Smile esthetics are a crucial aspect of facial attractiveness, playing a central role in social interactions. Dental students’ perception of smiling esthetics may evolve as they progress through their education and clinical exposure. This study aimed to investigate the influence of educational level on dental students’ perception of altered smile esthetics. **Methods**: A cross-sectional study was conducted among 410 undergraduate dental students across five academic years at the National and Kapodistrian University of Athens. Participants evaluated 22 digitally altered smile images, including single and combined esthetic discrepancies, using a visual analog scale (VAS). Perceived attractiveness scores were analyzed in relation to academic year, gender, and specific types of smile alterations. **Results**: The perception of smile attractiveness varied significantly across academic years for certain esthetic discrepancies, including central incisor length mismatch, midline diastema, and open gingival embrasures (*p* < 0.05). Clinical-year students (years 4–5) demonstrated a more critical assessment compared to preclinical students. Female students exhibited greater sensitivity to specific discrepancies, including fluorosis and reduced tooth lightness. The combination of a midline diastema, a gummy smile, and reduced lightness received the lowest attractiveness scores across all groups. **Conclusions**: The perception of altered smile esthetics among undergraduate dental students evolves throughout their education, although this progression does not follow a linear trajectory. Dental education appears to influence the perception of specific smile esthetic discrepancies, reflecting a selective influence on features. Clinical training appears to be a critical parameter of dental education, influencing the perception of smiling esthetics.

## 1. Introduction

Physical appearance plays a significant role in shaping human behavior and social interactions across different cultures and age groups [1]. Among facial features, the smile holds a key role in forming first impressions and fostering interpersonal relationships [2,3]. An attractive smile not only enhances facial esthetics but also contributes to an individual’s self-esteem and psychological well-being [4]. On the other hand, deficiencies in smile esthetics can adversely impact not only the smile itself but also the perception of overall facial attractiveness [5].

The importance of smiling esthetics has grown substantially in recent decades [6]. Patients are seeking an esthetically pleasing smile that resembles the esthetic standards presented in society and the media, where a beautiful smile is often associated with success [7,8]. To enhance smile esthetics, a comprehensive analysis of the components that determine a smile’s attractiveness is required [9,10].

The harmony and symmetry of an esthetic smile are influenced by various factors, each with a different level of importance [5]. These factors include dental alignment, tooth exposure, gingival display, smile arc, tooth proportions, the presence of a midline shift, axial inclination, buccal corridors, diastema, and tooth color [5,11,12,13,14]. While each factor can be considered individually, their combined influence contributes to the overall esthetic effect [5].

The perception of smile esthetics can vary based on geographic, ethnic, cultural, and demographic factors [15,16,17]. Dental professionals play a crucial role in helping patients make informed decisions about esthetic treatments by aligning clinical needs with patient preferences [18,19]. This responsibility requires a thorough understanding of the available scientific evidence, as well as the ability to balance this knowledge with patients’ perceptions and expectations [20].

Dental education and clinical experience play a significant role in shaping the perception of smile esthetics, enabling clinicians to identify esthetic discrepancies and recommend appropriate treatments [21,22,23]. Before beginning their training, dental students often lack the diagnostic skills necessary for a comprehensive assessment of smile esthetics [24]. As a result, understanding how the perception of smile esthetics evolves throughout dental education is crucial.

In the literature, numerous studies have been conducted to evaluate esthetic discrepancies, aiming to investigate how dentists and laypersons perceive smile esthetics [5,12,13,14,15]. One standard method for investigating smile esthetic discrepancies involves digitally simulating altered smile esthetics using photographs or portraits [12,13,14,15]. By modifying one or more components of the smile, their impact on the perceived attractiveness of the smile can be assessed.

The perception of smile esthetics by dental students has been explored in several studies [21,22,24,25,26]. However, the evidence regarding the effect of dental education on the perception of smiling esthetics remains scarce. Therefore, the present study aimed to assess the influence of educational level on dental students’ perception of altered smile esthetics. The research hypothesis was that there would be no difference in the perceived attractiveness of smiles among dental students at different educational levels.

## 2. Materials and Methods

**Figure 1:**
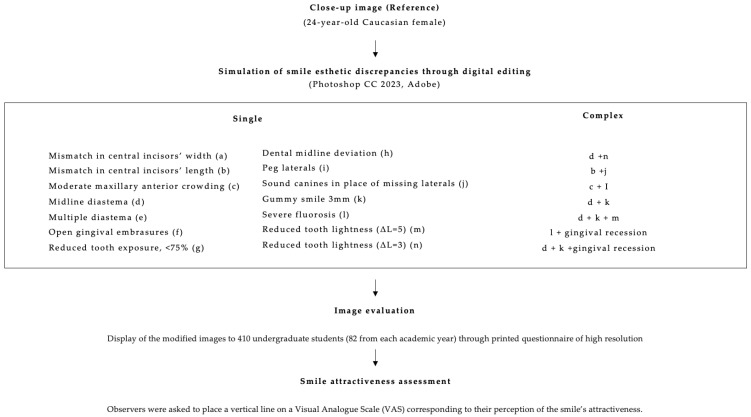
Flowchart illustrating the study methodology.

The present cross-sectional study was conducted at the Dental School of the National and Kapodistrian University of Athens, Greece. Ethical approval was granted by the Ethics Board of the Dental School, and the study was registered under the protocol number 395/20/2022. All data were collected anonymously, and confidentiality was maintained throughout the study. Informed consent was obtained from each participant before their enrollment in the study. A flowchart illustrating the study design is presented in Figure 1.

Based on previous studies, a power analysis was conducted using an online software (https://registrar.uiowa.edu/gpa-calculator, accessed on 23 September 2023, Power Calculator, University of Iowa, Iowa City, IA, USA), with an effect size of 0.60 at a conventional α level of 0.05 for a desired power (1−β) of 0.85. A total of 410 undergraduate dental students were randomly selected to participate in the present study, with 82 students recruited from each of the five academic years of the five-year dental program. In the current curriculum, training in smile esthetics is included as a course for third-year students before the start of their clinical training. It is subsequently reinforced through clinical practice during the fourth and fifth years of dental education.

**Figure 2:**
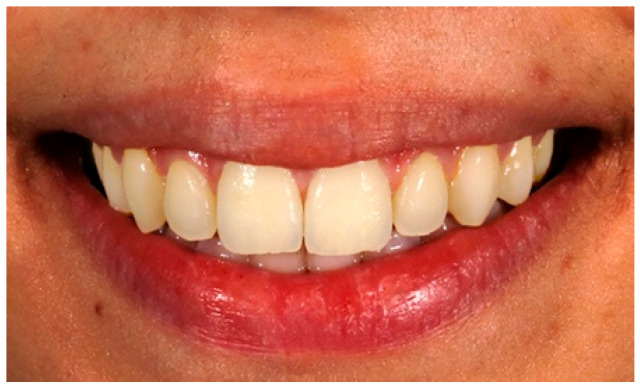
Digital image used as the control.

A 24-year-old Caucasian female with a high smile line and a good tooth alignment, consistent with Rufenacht’s tooth-papilla-gingival ideals and proportions, was selected to serve as the model for the present study [27]. A high-resolution digital close-up image of the model in a full smile, showing her teeth, was captured using a Canon EOS 80D digital camera (Canon, Tokyo, Japan) with a 100 mm macro lens (IS, USM, Tokyo, Japan) and two Canon 270EX II wireless flashes (Figure 2). The initially captured image served as the control.

**Figure 3:**
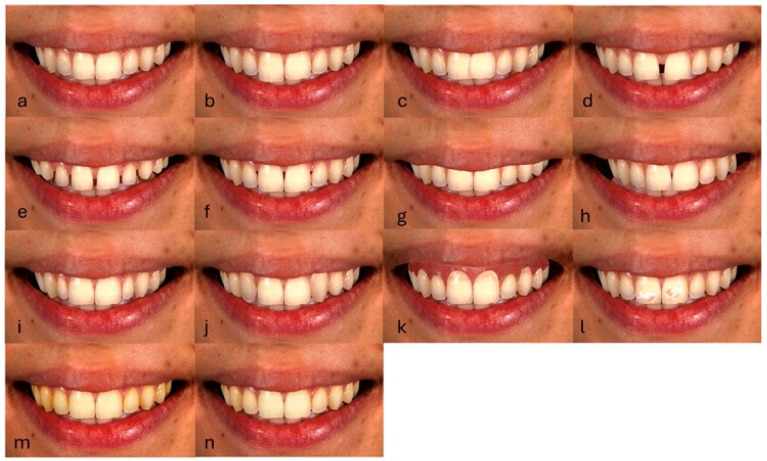
Modified images simulating a single esthetic discrepancy.

Subsequently, the control image was modified using a digital image editing software (Adobe Photoshop CS 2023, Adobe, San Jose, CA, USA). In total, 21 modified images were created, simulating a series of altered smile esthetics. Of the modified images, fourteen (14) included a single esthetic discrepancy (Figure 3): mismatch in central incisors’ width (2 mm) (a), mismatch in central incisors’ length (1 mm) (b), moderate maxillary anterior crowding (c), midline diastema (1.5 mm) (d), multiple diastema (e), open gingival embrasures (f), reduced tooth exposure (<75%) (g), dental midline deviation (4 mm) (h), peg laterals (i), sound canines in place of missing lateral incisors (j), excessive gingival display (gummy smile) (3 mm) (k), severe fluorosis (l), reduced tooth lightness (ΔL = 5) (m), and reduced tooth lightness (ΔL = 3) (n).

**Figure 4:**
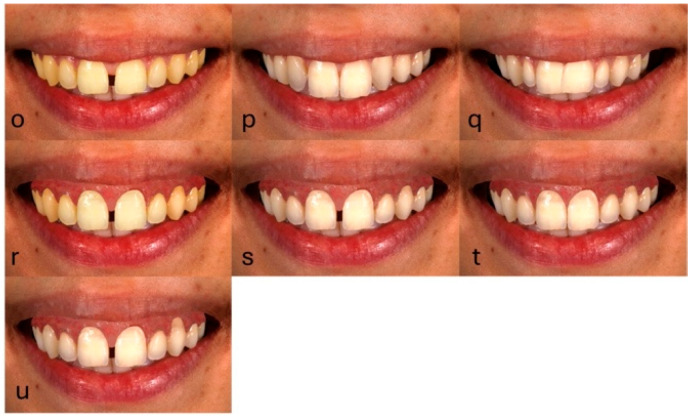
Modified images simulate multiple esthetic discrepancies.

For the remaining seven (7) images a combination of smile components was altered, resulting in the following complex esthetic discrepancies (Figure 4): midline diastema and reduced tooth lightness (o); sound canines in place of lateral incisors and central incisor with an increased length (p); moderate maxillary anterior crowding and peg laterals (q); midline diastema and gummy smile (r); midline diastema, gummy smile, and reduced tooth lightness (ΔL = 5) (s); gummy smile and gingival recession on canine (t); and midline diastema, gummy smile, and gingival recession (u).

Each participant completed the study through an in-person interview session. The age and gender of each student were recorded at the beginning of the survey. Participants were blinded to the digital manipulations of the images. The control and modified images were displayed in a randomized sequence at the actual size under standardized lighting conditions, ensuring that they were not exposed to direct sunlight.

Each image was viewed for only 10 s. Everyone rated the images without conferring with the others. The evaluations were performed using a visual analog scale (VAS) ranging from 0 (extremely unattractive) to 100 (extremely attractive). For each image, participants were asked to mark a vertical line on the VAS in response to the question, “How attractive do you consider the presented smile?” The distance from the start of the line to the mark represented the participant’s perceived smile attractiveness score. Scores could not be altered after submission.

### Statistical Analysis

Descriptive statistics were used to present the data. Differences in visual analog scale (VAS) scores between the control and each of the 21 altered images were recorded and analyzed. Numerical outcomes were calculated based on means and standard deviations. Preliminary analysis, including normal probability plots and the Anderson–Darling test for normality, revealed significant deviations from a normal distribution, primarily due to the presence of outliers across most variables. Given this deviation, nonparametric statistical methods were employed to evaluate differences in smile attractiveness among the studied groups. The Wilcoxon signed-rank test was used to assess these differences based on the age group, gender, and observer type. The analysis was conducted using the SPSS software (version 27, IBM, Chicago, IL, USA), with a significance level set at *p* < 0.05.

## 3. Results

**Figure 5:**
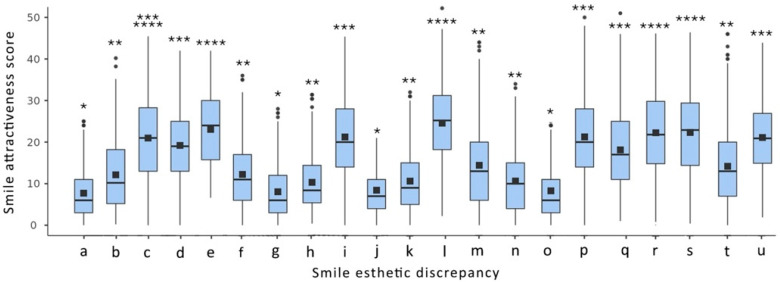
Boxplot of smile attractiveness score differences between each smile discrepancy and the control, along with the statistically significant differences (*p* < 0.05), based on the total sample of dental students. (Dots indicate outliers; a different number of asterisks (*) represent statistically significant differences between the investigated groups).

The students had a mean age of 21.1 years (SD = 2), with ages ranging from 18 to 32 years. Among them, 185 were male and 225 were female. First-year students had an average age of 18.3 years (SD = 1.8), comprising 45 females and 37 males. Second-year students averaged 19.2 years (SD = 1.9), comprising 44 females and 38 males. Third-year students had a mean age of 20.4 years (SD = 2.2), comprising 44 females and 38 males. Fourth-year students averaged 21.3 years (SD = 2.1), comprising 47 females and 35 males. Finally, the fifth-year students had an average age of 22.5 years (SD = 2.5), comprising 45 females and 37 males. As 95% of the students were born and raised in Greece, nationality was not included in the analysis. A boxplot illustrating smile attractiveness scores and the statistically significant differences (*p* < 0.05) between the evaluated groups for each smile discrepancy, based on the total sample of dental students, is presented in Figure 5.

The mean differences in smile attractiveness scores between the control and each evaluated discrepancy ranged from 7.71 (SD = 6.52) to 23.5 (SD = 10.26). Discrepancies such as mismatched central incisor width, reduced tooth exposure, and dental midline deviation resulted in significantly lower attractiveness scores (*p* < 0.05) compared to the other evaluated smile esthetic discrepancies. Complex, smile esthetic discrepancies, including a combination of a midline diastema and a gummy smile and a combination of a midline diastema, a gummy smile, and reduced tooth lightness (ΔL = 5), were perceived as significantly less attractive (*p* < 0.05) than all other simulated smile esthetic discrepancies.

**Figure 6:**
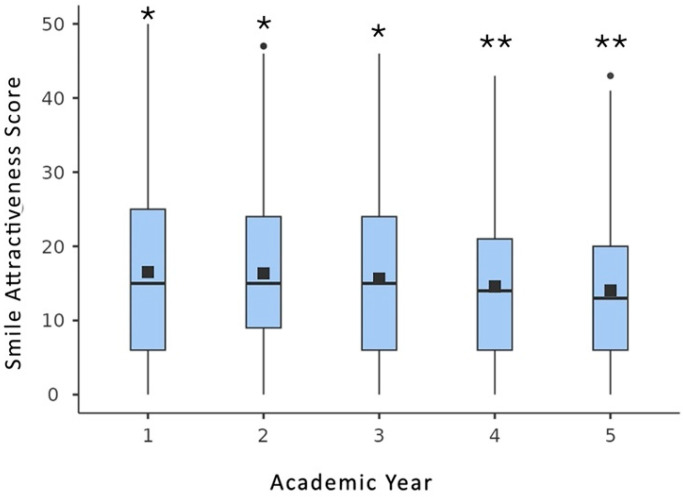
Boxplot of smile attractiveness score differences between each smile discrepancy and the control, along with the statistically significant differences (*p* < 0.05) in overall mean smile attractiveness scores among students of each academic year. (Dots indicate outliers; a different number of asterisks (*) represent statistically significant differences between the investigated groups).

A boxplot of smile attractiveness scores, along with the statistically significant differences (*p* < 0.05) in overall mean smile attractiveness scores among students of each academic year, is presented in Figure 6.

When considering the overall mean smile attractiveness scores for the simulated esthetic discrepancies, significant differences were found among dental students across different academic years (*p* < 0.05).

Mean smile attractiveness scores with standard deviations, as well as the statistically significant differences (*p* < 0.05) for each smile esthetic discrepancy among students by academic year, are presented in Table 1.

For smile esthetic discrepancies, such as mismatched central incisor length, a gummy smile, a midline diastema, and an open gingival embrasure, significant differences (*p* < 0.05) were found among students in different academic years. However, no statistically significant differences (*p* > 0.05) were observed across academic years for other discrepancies, including mismatched central incisor width, dental midline deviation, and the presence of sound canines in place of missing lateral incisors.

Mean smile attractiveness scores, standard deviations, and statistically significant gender differences (*p* < 0.05) for each simulated smile esthetic discrepancy are summarized in Table 2.

**Figure 7:**
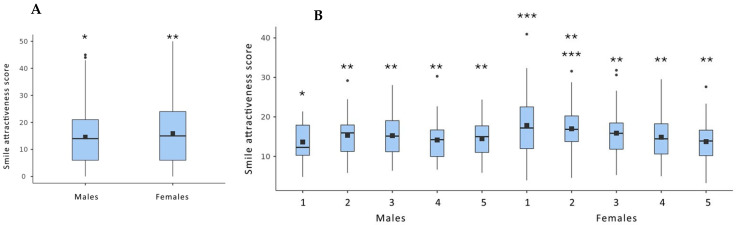
(**A**) Boxplot showing the differences in smile attractiveness scores between overall smile discrepancies and the control, including statistically significant differences (*p* < 0.05), grouped by student gender. (**B**) A. boxplot showing the differences in smile attractiveness scores between overall smile discrepancies and the control, including statistically significant differences (*p* < 0.05), grouped by student gender for each academic year. (Dots indicate outliers; a different number of asterisks (*) represent statistically significant differences between the investigated groups).

A significant difference in perceived smile attractiveness scores was observed between male and female participants (Figure 7A). However, this difference diminished among students in higher academic years. (Figure 7B).

A statistically significant difference (*p* < 0.05) in mean smile attractiveness scores between male and female participants was observed only for the following simulated smile esthetic discrepancies: moderate maxillary anterior crowding, open gingival embrasures, severe fluorosis, reduced tooth lightness, mismatch in central incisors’ length along with sound canines in place of missing laterals, and a midline diastema combined with a gummy smile and gingival recession. In these cases, female students rated the smiles as significantly less attractive than male students.

## 4. Discussion

In contemporary dental practice, esthetic dentistry is rapidly evolving. Dental schools play a crucial role in ensuring the graduation of experienced clinicians who can make accurate diagnoses and develop effective treatment plans. Therefore, it is essential to educate dental students on the principles of dentofacial esthetics [28].

The present cross-sectional study aimed to evaluate the impact of educational level on the perception of altered smile esthetics. The null hypothesis was partially rejected, as differences in perceived smile attractiveness were observed among students at different stages of education, but only for a part of the evaluated esthetic discrepancies. Similarly, gender-related differences were identified, but these were also limited to specific aspects of altered smile esthetics.

Although numerous studies have examined the perception of smiling among dental students, making an objective comparison between the findings of the present study and those of previous research is challenging. This can be attributed to the variability in data collection instruments, analytical methods, the specific smile features evaluated, and the inclusion of diverse sociocultural parameters. The outcome of the present research aligns with previous studies, which have demonstrated that the perception of smile esthetics is developed throughout dental education [28].

In the present study, a difference in the perception of smile esthetics was observed between pre-clinical dental students (in the first three years of study) and those in the fourth and fifth years, who had progressed through their clinical training. This could be explained by the clinical exposure gained after the third year, combined with the knowledge that was acquired during the previous years of study. These findings are consistent with previous studies, which report that final-year students demonstrate significantly different perceptions of altered smile esthetics, supporting the notion that the perception of smile esthetics develops progressively throughout dental education [24,25,29]. The one-hour esthetics course received by third-year students appears to be insufficient to significantly influence their perception of smile esthetics.

The outcomes of the present study are partially in agreement with those of a previous study, as dental education appears to influence the perception of each smile discrepancy differently [22,23,29]. As students’ progress through their studies, they may become critical depending on the specific esthetic feature being assessed [29]. This variation may also be explained by an increased understanding of patients’ needs and expectations regarding smile esthetics, gained through clinical training [30].

In the present study, as well as in similar studies in the literature, the perception of confident smile esthetic discrepancies appeared to remain unchanged or follow an inconsistent progression across academic years [21,22,23]. Additionally, some discrepancies, such as the width of the central incisors and dental midline deviation, were not perceived at all, regardless of the students’ academic level. Therefore, the effectiveness of the dental education program may need to be reconsidered [31]. Specifically, at the end of their undergraduate studies, students must develop the ability to assess smile esthetics critically. However, according to the literature, this skill is often fully developed during postgraduate training [32].

Regarding the gender variable, our analysis revealed that, for some of the evaluated esthetic discrepancies, female students demonstrated greater sensitivity in perceiving specific components compared to their male counterparts. The generally greater interest seen in women may explain this outcome, which tends to show in their appearance [32,33,34,35]. The difference in perceived smile attractiveness between the genders appeared to be reduced at higher academic levels. The observed gender differences in esthetic perception may have implications for clinical practice. Female students demonstrated greater sensitivity to certain smile discrepancies. This heightened perception may translate into more nuanced esthetic evaluations and treatment planning when these individuals become clinicians. Conversely, male students may benefit from targeted training to enhance their attention to subtle esthetic details. The existing literature remains inconclusive regarding the influence of gender on dental students’ ability to detect esthetic discrepancies in smile esthetics. The studies showed no difference or found that male students had a better perception of dental esthetics compared to females [21,22,23]. Also, one study found that male students exhibited a better perception of altered smile esthetics compared to females [5]. It remains unclear whether these inconsistencies are due to variations in geographic location and behavioral cultural context or differences in study methodology [36]. These findings suggest that dental education curricula could be tailored to address perceptual differences, ensuring a consistent standard of care regardless of the clinician’s gender. Moreover, understanding these variations could be helpful in interdisciplinary team discussions, treatment planning, and aligning esthetic goals with patient expectations

In the present study, a series of digitally modified images simulating both individual and complex smile discrepancies was used. To the best of the authors’ knowledge, this is the first study in the literature to assess esthetic perception by simultaneously altering multiple components of the smile. Additionally, a large sample of dental students from all academic years has been implemented.

This study has several limitations that should be considered when interpreting the results. Only one of the existing approaches to evaluating smile esthetics was implemented, without incorporating variations in the degree of alteration. As a result, the impact of the severity of each smile discrepancy on perceived smile attractiveness could not be assessed. Furthermore, only a limited range of smile esthetic discrepancies was evaluated, as participant eye fatigue had been taken into consideration [12].

Smile attractiveness is inherently subjective, and differences may occur even when the same observer evaluates the same image multiple times. Moreover, several factors such as the surrounding environment, viewing angle, and the observer’s mood can influence the assessment [5]. In the present study, the reliability of the evaluation method was not assessed, as this would have required a further increase in the number of images presented.

Although the visual analog scale (VAS) has been widely used in evaluating facial esthetics, it is not without limitations. Raters often tend to distribute their responses across the entire scale, avoiding the extremes of the anchor points [37]. Additionally, observers may be unable to make equally discriminative judgments across the entire scale range [38]. Nevertheless, the evaluation methods applied in this study have been extensively used in similar research and are recognized in the scientific literature as a valid and reliable tool [5].

Another limitation of the present study was that students from different years were included, rather than investigating a specific group of students at repeated points in time throughout their undergraduate studies. However, all participants were from the same dental school and were enrolled in the same program of study. Additionally, the study did not explore the effects of nationality or socio-economic status, as investigating such correlations would be highly complex and would require a significantly larger and more diverse sample from multiple countries and social backgrounds. Given that only students from one academic institution were included, the findings of the present study need to be validated by future research that includes students from dental schools around the world. Furthermore, to better simulate clinical reality, future studies could use portrait images or 3D virtual representations of models, acquired by face scanners, instead of a close-up image.

## 5. Conclusions

The perception of altered smile esthetics among undergraduate dental students evolves throughout their education, although this progression does not follow a linear trajectory. Dental education appears to influence the perception of specific smile esthetic discrepancies, reflecting a selective influence on features. Clinical training appears to be a critical parameter of dental education, influencing the perception of smiling esthetics. Dental education must provide the appropriate background for evaluating smile esthetics based on the available scientific evidence, enabling dental students to properly develop their smile perception.

## Figures and Tables

**Table 1:** Difference in perceived smile attractiveness scores of altered images compared to the control, along with standard deviations, among undergraduate students by academic year. The same superscript does not indicate a significant difference. The different letters indicate significant differences.

Smile Esthetic Discrepancy		Academic Year				
		1	2	3	4	5
**Single**	Mismatch in central incisors’ width (a)	7.96 (8.09) ^a^	7.60 (5.68) ^a^	7.86 (5.77) ^a^	6.92 (6.41) ^a^	8.23 (6.49) ^a^
	Mismatch in central incisors’ length (b)	13.27 (9.06) ^a^	12.57 (8.63) ^a^	11.96 (8.23) ^a,b^	11.23 (9.09) ^a,b^	10.61 (7.76) ^b^
	Moderate maxillary anterior crowding (c)	23.01 (11.54) ^a^	25.75 (8.54) ^a^	24.22 (9.97) ^a^	19.77 (10.06) ^b^	16.82 (10.12) ^b^
	Midline diastema (d)	21.50 (11.52) ^a^	20.58 (8.60) ^a^	18.21 (9.44) ^b^	18.65 (10.16) ^b^	17.06 (8.61) ^b^
	Multiple diastema (e)	23.37 (12.11) ^a^	22.13 (10.60) ^a^	20.56 (9.50) ^a,b^	19.12 (9.03) ^b^	19.70 (9.73) ^b^
	Open gingival embrasures (f)	14.86 (10.19) ^a^	12.67 (8.20) ^b^	11.16 (7.86) ^b,c^	12.71 (7.73) ^b^	9.77 (7.10) ^c^
	Reduced tooth exposure, <75% (g)	9.53 (7.97) ^a^	9.51 (7.79) ^a^	7.81 (6.40) ^a,b^	6.52 (6.36) ^b^	6.91 (6.05) ^b^
	Dental midline deviation (h)	10.75 (8.92) ^a^	9.47 (6.69) ^a,b^	7.88 (6.00) ^b^	10.53 (9.18) ^a^	11 (8.62) ^a^
	Peg laterals (i)	23.80 (11.95) ^a^	23.25 (9.45) ^a^	21.27 (9.71) ^a,b^	20.02 (10.72) ^b^	17.82 (9.08) ^c^
	Sound canines in place of missing laterals (j)	8.93 (7.42) ^a^	8.15 (6.64) ^a^	9.22 (6.72) ^a^	8.10 (7.32) ^a^	7.73 (5.22) ^a^
	Gummy smile 3 mm (k)	9.37 (8.14) ^a^	9.65 (8.02) ^a^	11.87 (8.79) ^b^	10.86 (7.72) ^a,b^	11.32 (6.64) ^b^
	Severe fluorosis (l)	18.52 (10.73) ^a^	17.37 (8.80) ^a^	18.16 (8.75) ^a^	15.40 (9.29) ^b^	16.17 (8.90) ^b^
	Reduced tooth lightness (ΔL = 5) (m)	18.07 (11.43) ^a^	17.38 (9.62) ^a^	13.71 (8.66) ^b^	11.55 (10.22) ^c^	11.38 (9.57) ^c^
	Reduced tooth lightness (ΔL = 3) (n)	10.81 (8.97) ^a^	10.45 (7.73) ^a^	7.11 (6.70) ^b^	7.53 (6.44) ^b^	5.51 (4.39) ^c^
**Complex**	d + n (o)	23.93 (11.77) ^a^	23.25 (9.45) ^a^	21.27 (9.71) ^a,b^	20.02 (10.72) ^b^	17.82 (9.08) ^c^
	b + j (p)	9.97 (9.16) ^a^	10.78 (8.06) ^a^	11.08 (8.58) ^a^	10.82 (7.93) ^a^	10.51 (7.56) ^a^
	c + i (q)	19.11 (10.98) ^a^	19.16 (8.56) ^a^	21.31 (8.96) ^a^	19.26 (9.38) ^a^	16.83 (8.98) ^b^
	d + k (r)	23 (11.93) ^a^	22.02 (9.04) ^a^	23.22 (8.72) ^a^	21.67 (8.91) ^a^	21.86 (8.82) ^a^
	d + k + m (s)	24.05 (11.33) ^a^	25.10 (9.14) ^a^	23.91 (10.31) ^a^	20.67 (10.32) ^b^	21.52 (9.62) ^b^
	k + gingival recession (t)	12.46 (10.17) ^a^	14.18 (9.26) ^a^	15.36 (10.30) ^b^	14.61 (9.92) ^b^	14.47 (8.63) ^b^
	d + k + gingival recession (u)	22.76 (11.79) ^a^	22.35 (8.71) ^a^	22.32 (9.26) ^a^	21.35 (9.47) ^a^	21.46 (9.07) ^a^

**Table 2:** Difference in perceived smile attractiveness scores of altered images compared to the control, along with standard deviations for each smile esthetic discrepancy across genders. The same superscript does not indicate a significant difference. The different letters indicate significant differences.

Smile Esthetic Discrepancy		Gender	
		Male	Female
**Single**	Mismatch in central incisors’ width (a)	8.21 (6.89) ^a^	6.89 (5.79) ^a^
	Mismatch in central incisors’ length (b)	10.94 (7.58) ^a^	12.52 (9.09) ^a^
	Moderate maxillary anterior crowding (c)	15.63 (8.36) ^a^	18.02 (9.80) ^b^
	Midline diastema (d)	18.75 (8.80) ^a^	19.47 (10.38) ^a^
	Diastema (multiple) (e)	19.80 (9.40) ^a^	21.68 (10.79) ^a^
	Gingival recession (Canine) (f)	13.15 (8.87) ^a^	14.79 (10.09) ^a^
	Open gingival embrasures (g)	10.94 (6.77) ^a^	13.01 (9.19) ^b^
	Reduced tooth exposure, <75% (h)	7.92 (6.36) ^a^	8.14 (7.43) ^a^
	Dental midline deviation (i)	10.32 (8.12) ^a^	9.69 (7.98) ^a^
	Peg laterals (j)	10.32 (8.12) ^a^	9.68 (7.98) ^a^
	Sound canines in place of missing laterals (k)	7.62 (6.29) ^a^	8.91 (6.90) ^a^
	Gummy smile (l)	10.70 (7.87) ^a^	10.56 (7.95) ^a^
	Severe fluorosis (m)	20.58 (10.39) ^a^	22.71 (10.50) ^b^
	Reduced tooth lightness (ΔL = 5) (n)	12.47 (9.44) ^a^	15.59 (10.67) ^b^
	Reduced tooth lightness (ΔL = 3) (o)	7.36 (5.49) ^a^	8.83 (8.11) ^a^
**Complex**	d + n	20.24 (9.08) ^a^	21.87 (11.06) ^a^
	k + b	9.48 (7.27) ^a^	11.33 (8.72) ^b^
	c + j	18.47 (8.68) ^a^	19.53 (9.91) ^a^
	d + l	22.31 (9.62) ^a^	22.38 (9.51) ^a^
	d +l + o	22.42 (9.95) ^a^	23.42 (10.44) ^a^
	l + f	12.46 (10.17) ^a^	14.18 (9.26) ^a^
	d + l +f	20.58 (10.39) ^a^	22.71 (10.50) ^b^

## Data Availability

The data presented in this study are available upon reasonable request from the corresponding author.
